# A Novel Multivariate Sample Entropy Algorithm for Modeling Time Series Synchronization

**DOI:** 10.3390/e20020082

**Published:** 2018-01-24

**Authors:** David Looney, Tricia Adjei, Danilo P. Mandic

**Affiliations:** Department of Electrical and Electronic Engineering, Imperial College London, London SW7 2AZ, UK

**Keywords:** multivariate sample entropy, time series synchronization, structural complexity

## Abstract

Approximate and sample entropy (AE and SE) provide robust measures of the deterministic or stochastic content of a time series (regularity), as well as the degree of structural richness (complexity), through operations at multiple data scales. Despite the success of the univariate algorithms, multivariate sample entropy (mSE) algorithms are still in their infancy and have considerable shortcomings. Not only are existing mSE algorithms unable to analyse within- and cross-channel dynamics, they can counter-intuitively interpret increased correlation between variates as decreased regularity. To this end, we first revisit the embedding of multivariate delay vectors (DVs), critical to ensuring physically meaningful and accurate analysis. We next propose a novel mSE algorithm and demonstrate its improved performance over existing work, for synthetic data and for classifying wake and sleep states from real-world physiological data. It is furthermore revealed that, unlike other tools, such as the correlation of phase synchrony, synchronized regularity dynamics are uniquely identified via mSE analysis. In addition, a model for the operation of this novel algorithm in the presence of white Gaussian noise is presented, which, in contrast to the existing algorithms, reveals for the first time that increasing correlation between different variates reduces entropy.

## 1. Introduction

Multivariate data analysis tools are essential for characterizing the interaction between the variates of complex systems; applications are wide-ranging and include those in biology [1], climatology [2] and finance [3]. Standard methods for estimating interdependencies between multiple data channels are almost invariably linear; typical examples are cross-correlation and coherence (correlation in the frequency domain). More advanced methods, such as Granger causality [4], offer insight into the temporal ordering of interactions and are widely used in, for example, neuroscience applications, for which directionality information is of value. Partial directed coherence extends the concept of Granger causality to the frequency domain [5].

While progress has been made on nonlinear extensions of the above second-order algorithms, information-theoretic measures, such as mutual information and transfer entropy [6], are intrinsically suited to cater for higher-order interactions. Traditional entropy methods are limited by their requirements for large numbers of samples and sensitivity to noise [7]. To this end, the approximate entropy (AE) algorithm [7] was developed to provide a statistically valid measure of entropy for real-world univariate data that may be noisy and short. It represents the probability that similar patterns (delay vectors—DVs) in a time series will remain similar once the pattern lengths are increased (extended DVs), thereby providing a natural measure of the *regularity* of a time series. An extension, the sample entropy (SE) algorithm [8], improves the bias issues experienced by AE by omitting self-matches from the similarity calculation stage. In general, a stochastic time series is characterized by high AE/SE, while a regular signal exhibits low AE/SE. Additionally, multiscale extensions of the algorithm (multiscale sample entropy—MSE) [9,10] have expanded its capabilities beyond regularity estimation to evaluating the structural richness of a signal-generating system, its *complexity*. Both the SE and MSE algorithms have been widely used in physiological applications [11,12,13].

Recently, two multivariate extensions of the SE and MSE algorithms have been developed to evaluate the regularity and complexity for any number of data channels in a rigorous and unified way [14,15]. The key difference between the two multivariate algorithms is the manner in which multivariate DVs are extended. In the first extension, termed the naïve method [14], extended DV subspaces are generated for each variate, and self-matches are computed for each subspace separately. A shortcoming of the naïve method is its failure to account for inter-channel couplings, such as correlation between the variates. This was reflected in the “full method” [15]. Critically, a multivariate MSE (mMSE) method that caters fully for cross-channel dynamics enables the modeling of any complexity that exists between the variates, offering greater insight for physical systems that are typically multivariate and correlated. In [15], the benefits of a multivariate approach over univariate algorithms were demonstrated for applications spanning the categorization of wind regimes and the analysis of human gait recordings.

Despite the clear improvements of the full multivariate method over existing work, there are several concerns regarding its operation. Firstly, the way in which the DVs are extended is such that distances are directly calculated between elements of the different variates, potentially obscuring the physical meaning of the analysis for heterogeneous data. Secondly, such inconsistencies in the alignment of extended DVs can lead to inaccuracies. Finally, empirical results obtained using the full method for white Gaussian noise (WGN) and 1/f noise imply that regularity decreases and complexity increases with increasing correlation. However, viewing increased correlation between the variates as a decrease in cross-channel regularity is not consistent with physical intuition, motivating this work. It must also be noted that it has been suggested that the size of the time delay employed in the SE algorithm can have an effect on the computed entropy [16]; however, for the purposes of consistency across all analyses reported in this study, a unity time delay is employed.

To address these concerns, we propose a novel multivariate sample entropy (mSE) and its multiscale extension for complexity analysis (mMSE). At the core of the algorithm is our novel treatment of multivariate DVs, which ensures (i) element-by-element distances are not computed directly between different variates, and (ii) alignments between multivariate DVs remain consistent and independent of the number of data channels. Simulation results for WGN illustrate how the proposed algorithm interprets increased correlation between the variates as an increase in regularity—a missing result that is in agreement with physical intuition. To support this, we have derived a model for the performance of the algorithm in the presence of bivariate WGN; its numerical outcomes are in agreement with simulations. It is furthermore shown via a random alignment operation applied to the variates that this makes it possible to comprehensively distinguish between within- and cross-channel dynamics, thus providing additional insight into inter- versus intra-modal properties.

The algorithm is validated on synthetic and real data for biological applications. Through simulations, we reveal a previously unstudied feature of multivariate SE algorithms—their ability to detect synchronized regularity dynamics. Unlike other measures of synchronization, which assume temporal locking of phase information (phase synchrony [17,18]) or the existence of a functional mapping between the variates (generalized synchrony [19]), synchronized regularity can exist between time series that are generated by independent processes.

## 2. Sample Entropy

Motivated by the shortcomings of standard entropy measures for short and noisy time series, the approximate entropy technique was introduced in [7]. It characterizes the likelihood that similar patterns within a time series, the signal DVs, will remain similar when the pattern lengths are increased. A robust extension, which neglects self-matches, called the SE, has been developed [8] and is described below:

1. For lag τ and embedding dimension *m*, generate DVs:
  (1) $$X_{m}\left(i\right)=\left[x_{i},x_{i+1},\ldots ,x_{i+\tau (m-1)}\right]$$
  where $i=1,2,\ldots ,\left(N-\tau (m-1)\right)$.
2. For a given DV, $X_{m}\left(i\right)$, and a threshold, *r*, count the number of instances, $\Phi_{m}\left(i,r\right)$, for which $d\{X_{m}\left(i\right),X_{m}\left(j\right\}\leq r$, i≠j, where d{·} denotes the maximum norm.
3. Define the frequency of occurrence as
  (2) $$\Phi_{m}\left(r\right)=\frac{1}{N-\tau (m-1)+1}\sum_{i=1}^{N-\tau (m-1)+1}\Phi_{m}\left(i,r\right)$$
4. Extend the embedding dimension (m→m+1) of the DVs in step (1), and repeat steps (2) and (3) to obtain $\Phi_{m+1}\left(r\right)$.
5. The SE is defined as the negative logarithm of the values for different embedding dimensions, that is,
  (3) $$\mathrm{SE}\left(m,r,\tau \right)=-ln\left[\frac{\Phi_{m+1}\left(r\right)}{\Phi_{m}\left(r\right)}\right]$$

**Figure 1:**
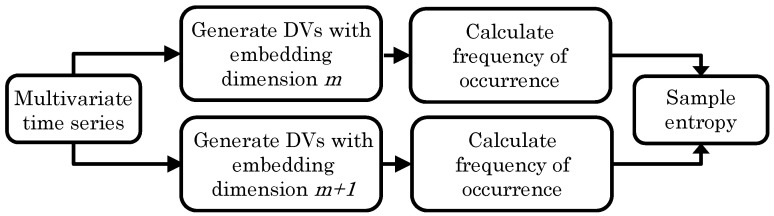
Block diagram of the multivariate sample entropy (mSE) algorithm.

In general, the less predictable or the more irregular a time series, the higher its SE. A block diagram of the algorithm is shown in Figure 1.

### 2.1. Multiscale Sample Entropy

The MSE calculates the SE over multiple time scales in the data [9,10]. A key insight is given by the fact that SE for WGN (which has no structure) decreases for increasing scale factors, while the SE for 1/f noise, which has a self-similar infinitely repeating behaviour (contains structure), remains constant with scale. In this way, the multiscale extension reveals long-range signal correlations—dynamics closely linked to the signal complexity. If the SE of a time series remains high over multiple scale factors, it is said to exhibit high complexity.

## 3. Multivariate Sample Entropy

### 3.1. Existing Algorithms

**Figure 2:**
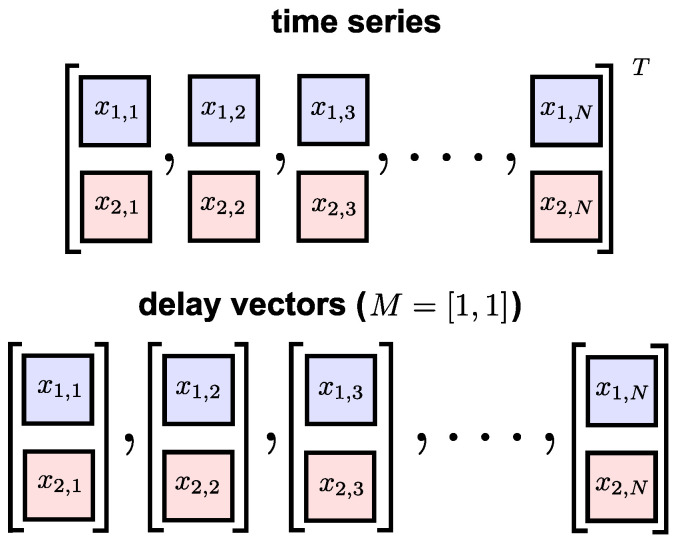
A bivariate time series (upper panel) and its delay vectors with embedding dimension M=[1,1] (lower panel).

Two multivariate extensions of the SE algorithm have been proposed in [14,15]. In both cases, the *un-extended* multivariate DVs are generated on the basis of the approach outlined by Cao et al. [20]. Given a *P*-variate time series, $x_{k,i}$ where k=1,…,P is the channel index and i=1,…,N is the sample index, the multivariate DVs are given by

(4) $$\begin{array}{ccc} X_{M}\left(i\right) & = & [x_{1,i},\ldots ,x_{1,i+\left(m_{1}-1\right)\tau_{1}},\\ & & x_{2,i},\ldots ,x_{2,i+\left(m_{2}-1\right)\tau_{2}},\\ & & \ldots ,\\ & & x_{P,i},\ldots ,x_{P,i+\left(m_{P}-1\right)\tau_{P}}] \end{array}$$

where $M=[m_{1},m_{2},\ldots ,m_{P}]$ is the multivariate embedding dimension vector and $ø=[\tau_{1},\tau_{2},\ldots ,\tau_{P}]$ is the multivariate time-lag vector. Figure 2 shows a bivariate time series and its multivariate DVs with embedding dimension M=[1,1].

**Figure 3:**
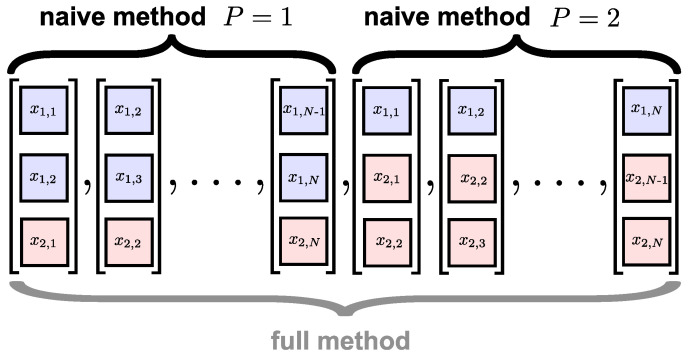
The *extended* multivariate delay vectors (DVs) of the multivariate DVs shown in Figure 2 using existing techniques. The *naïve method* [14] calculates the pair-wise distances within the two subspaces (P=1 and P=2) separately, while the *full method* [15] calculates the pair-wise distances across all the delay vectors.

To extend the multivariate DVs, the naïve approach in [14] generates *P* DV subspaces, in which the length of a specific variate is extended but other variates are left unchanged. The extended DV subspace for a variate *k* is given by

(5) $$\begin{array}{ccc} X_{M}^{e_{k}}\left(i\right) & = & [x_{1,i},\ldots ,x_{1,i+\left(m_{1}-1\right)\tau_{1}},\ldots ,\\ & & x_{k,i},\ldots ,x_{k,i+\left(m_{k}-1\right)\tau_{k}},x_{k,i+\left(m_{k}\right)\tau_{k}},\\ & & \ldots ,\\ & & x_{P,i},\ldots ,x_{P,i+\left(m_{P}-1\right)\tau_{P}}] \end{array}$$

for i=1,…,N and $e_{k}=e_{1},\ldots ,e_{P}$. For the bivariate case, this approach to DV extension is illustrated in Figure 3. Distances are then calculated between DV pairs in each of the *P* subspaces to obtain *P* estimates for the frequency of occurrence, and the final value is obtained by averaging the subspace estimates. The next steps of the algorithm are the same as for the univariate case. A significant shortcoming of the naïve method is that, by measuring distances between the DVs within separate subspaces, it does not cater fully for inter-channel couplings. This was illustrated in [15], where the naïve method could not distinguish between different degrees of correlated variates.

**Figure 4:**
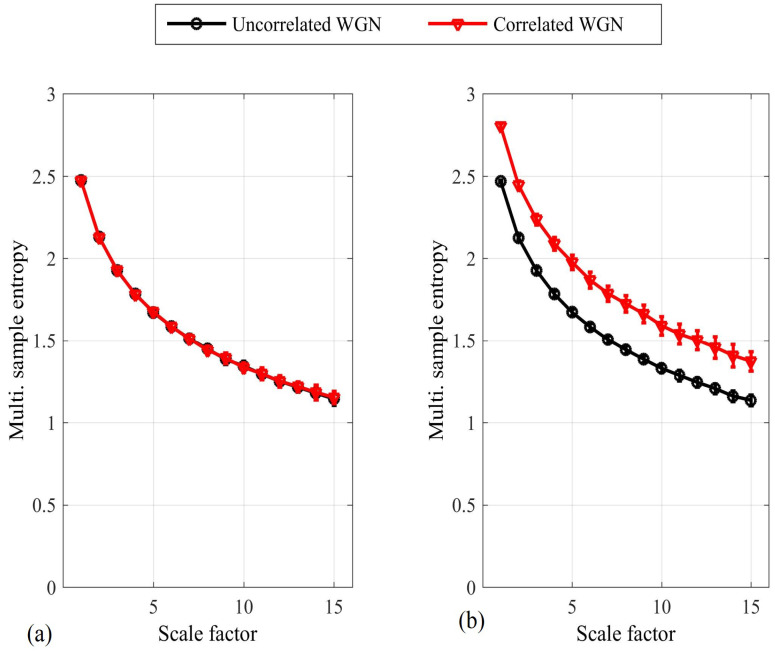
Multivariate sample entropy (mSE) estimate for bivariate white Gaussian noise (WGN), correlated and uncorrelated, using the naïve method [14] (**a**); and the full or existing method [15] (**b**). The error bars denote ± standard deviation. We observe that only the full/existing method can distinguish between correlated and uncorrelated variates.

To address these issues, the full method was proposed in [15], in which pair-wise distances are calculated between all DVs across all the subspaces, as described in Equation (5) and illustrated for the bivariate case in Figure 3. This enables the enhanced modeling of inter-channel couplings, as exemplified by its ability to distinguish between different degrees of correlation in the variates. The two approaches are compared in Figure 4 for bivariate WGN with a length of 5000 samples; the mSE parameters were M=[1,1], τ=[1,1] and r=0.15, each of the variates was standardized to unit variance and zero mean, and the correlation between the variates was ρ=0.95. We observe that while the naïve algorithm [14] cannot distinguish between correlated and uncorrelated WGN, the full algorithm [15] exhibits different SE values for each scale factor. As the full algorithm described in [15] represents the most recent multivariate extension to date; in the sequel we refer to it as the *existing method*.

Despite providing clear improvements over the previous mMSE algorithms, there are still issues regarding the existing method that need to be addressed:

1. Inability to cater adequately for heterogeneous data sources.
2. Inconsistencies in the alignment of DVs.
3. Counter-intuitive representation of multivariate dynamics.

Concerning item 1, in the standard method, the DV distances are directly calculated between elements of different variates. This is illustrated in Figure 3 for the bivariate case. If the variates are heterogeneous, that is, if they reflect different components of a biological system (e.g., cardiac and neural activity), then operations involving element-by-element comparisons will hinder the physical meaning of the analysis.

Concerning item 2 above, Figure 3 shows that one of the distances calculated for the extended DVs of a bivariate signal is between ${\left[x_{1,1},x_{1,2},x_{2,1}\right]}^{T}$ and ${\left[x_{1,2},x_{1,3},x_{2,2}\right]}^{T}$. In this instance, as is also the case in the operation of the univariate algorithm, the delays between corresponding elements are the same (+1 in this instance). However, when the distances are calculated between ${\left[x_{1,1},x_{1,2},x_{2,1}\right]}^{T}$ and ${\left[x_{1,1},x_{2,1},x_{2,2}\right]}^{T}$, this results in different delays between the DV elements: 0 $\left(\left\{x_{1,1},x_{1,1}\right\}\right)$, $-1\;(\left\{x_{1,2},x_{2,1}\right\})$ and +1 $\left(\left\{x_{2,1},x_{2,2}\right\}\right)$. This inconsistency in the alignment of the DV elements may cause contradictory results and affect the accuracy of the analysis.

Concerning item 3 above, Figure 4 illustrates that the multivariate SE increases across all scale factors for increasing correlation between the variates. Considering the algorithm output at the first scale factor only, the result indicates that regularity decreases with increasing correlation—a result that is not consistent with physical intuition. Instead, it is expected that as variates become more independent, it will be characterized by a decrease in regularity. Likewise, considering the algorithm output across all scale factors, the result indicates that complexity increases with increasing correlation. *No precise definition of complexity exists; however an intuitive expectation is that the number of required mathematical descriptors for a system should increase with complexity.* For this reason, it can be assumed that as the variates become more correlated/dependent, the system will be characterized by a decrease in complexity, the “complexity loss theory”.

### 3.2. Synchronized Regularity

The ability of multivariate SE algorithms to distinguish between coupled regularity dynamics has so far been confined to correlated variates [15] (see also Figure 4). In such studies, the couplings between the variates are static—they remain unchanged across time. Additionally, such relationships are adequately modeled by correlation estimation, not requiring the unique capabilities of a multivariate SE algorithm.

**Figure 5:**
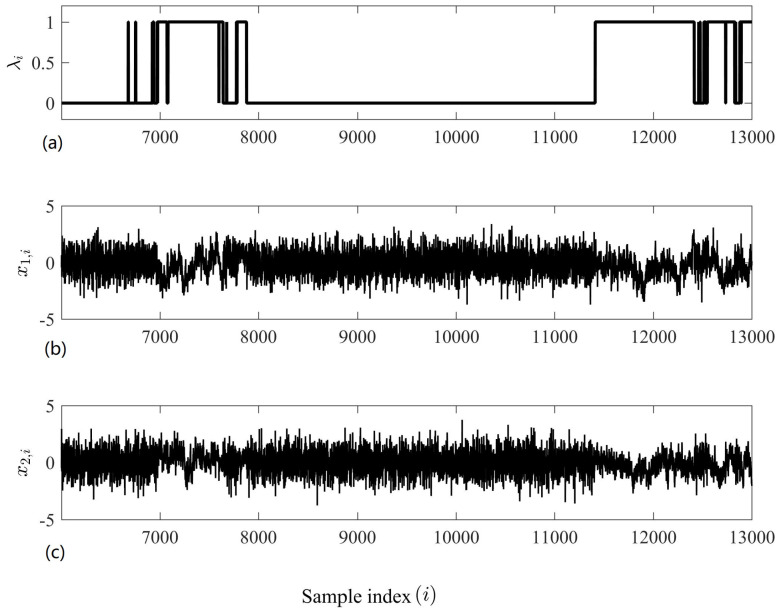
A bivariate time series exhibiting synchronized regularity. (**a**): The mixing parameter, which in this instance is the same for each variate. (**b**): The first variate. (**c**): The second variate. We observe that when $\lambda_{i}=1$ (Equations (6) and (7)), both variates exhibit 1/f-type structures (i.e., for i=7000,…,7500), and when $\lambda_{i}=0$ (Equations (6) and (7)), both variates exhibit WGN-type structures (i.e., for i=8000,…, 11,000).

We instead propose a multivariate benchmark test in which the *variates are generated from independent random processes but their regularity is synchronized*. In other words, the within-channel regularity changes at the same time within each variate. We consider the bivariate signal

(6) $$\begin{array}{cc} x_{1,i}= & \lambda_{1,i}^{\frac{1}{2}}v_{1,i}+{\left(1-\lambda_{1,i}\right)}^{\frac{1}{2}}u_{1,i} \end{array}$$

(7) $$\begin{array}{cc} x_{2,i}= & \lambda_{2,i}^{\frac{1}{2}}v_{2,i}+{\left(1-\lambda_{2,i}\right)}^{\frac{1}{2}}u_{2,i} \end{array}$$

where i=1,…,N, *N* is the number of samples, $\lambda_{1,i}$ and $\lambda_{2,i}\in \left[0,1\right]$ are mixing parameters, $v_{1,i}$ and $v_{2,i}$ are independent realizations of WGN (zero mean and unit variance), and $u_{1,i}$ and $u_{2,i}$ are independent realizations of 1/f noise (zero mean and unit variance). We note that no correlation, phase or generalized synchronization exists between the variates, as they are all independent. Instead, the parameter $\lambda_{i}$ enforces synchronized regularity; when $\lambda_{1,i}=\lambda_{2,i}=0$, the SE in both variates is low as each contains 1/f noise, and when $\lambda_{1,i}=\lambda_{2,i}=1$, the SE in both variates is high as each contains WGN. A dynamic mixing parameter can be generated by

(8) $$\lambda_{k,i}=\left\{\begin{array}{c} 1\;\;\;\mathrm{if}\;\;\;z_{i}\left(\beta \right)>0\\ 0\;\;\;\mathrm{if}\;\;\;z_{i}\left(\beta \right)<0 \end{array}\right.$$

where k∈{1,2}, i=1,…,N and $z_{i}\left(\beta \right)$ is a random noise time series with a spectral distribution that decays at rate β. When β=0, the time series is WGN; when β=−1, it is 1/f noise; and when β=−2, it is Brownian noise. Figure 5 shows a bivariate time series generated using the dynamic mixing model described by Equations (6)–(8) with β=−2; we observe that the within-channel regularities are synchronized.

**Figure 6:**
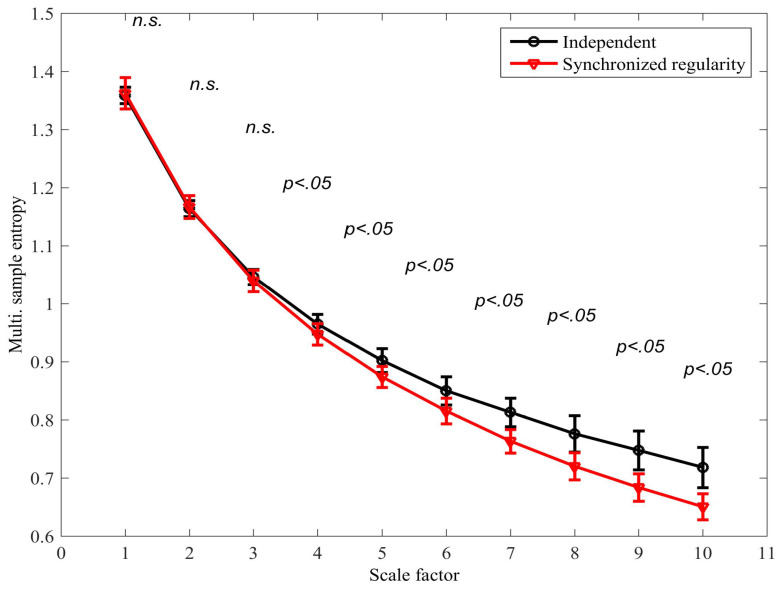
Multivariate multiscale sample entropy (mMSE) extension using the existing algorithm for signals with (i) independent mixing parameters, and (ii) synchronized mixing parameters; see model described by Equations (6)–(8). The error bars denote ± standard deviation. A two-tailed two-sample *t*-test was applied to determine the degree of separation between the two scenarios at each scale factor; instances in which the distributions were found to overlap are denoted by “not-significant” (n.s.).

The existing mMSE algorithm was applied to distinguish between the scenarios in which (i) the variates are independent, that is, $\lambda_{1,i}\neq \lambda_{2,i}$; and (ii) the regularity of the variates is synchronized, that is, $\lambda_{1,i}=\lambda_{2,i}$. In both scenarios, β=−1.6 (see Equation (8)) was used to generate the mixing parameters. The signal length was *N* = 15,000 samples; r=0.4 and M=[1,1]. Figure 6 shows the mMSE for each scenario; a two-tailed two-sample *t*-test was applied to determine the degree of separation at each scale factor (38 degrees of freedom). We observe that a statistically significant separation was determined at scale factor 4 (p<0.05) and at all subsequent scale factors. It is also noted that the *synchronized regularity caused a decrease in the mSE*; this result is in agreement with physical intuition but contradicts results obtained with the same algorithm for correlated variates (see Figure 4b), where coupled regularity dynamics was found to increase the multivariate SE. As real-world processes can be expected to exhibit different forms of coupled regularity dynamics simultaneously, this compromises the accuracy of the algorithm in applications.

### 3.3. The Proposed Algorithm

**Figure 7:**
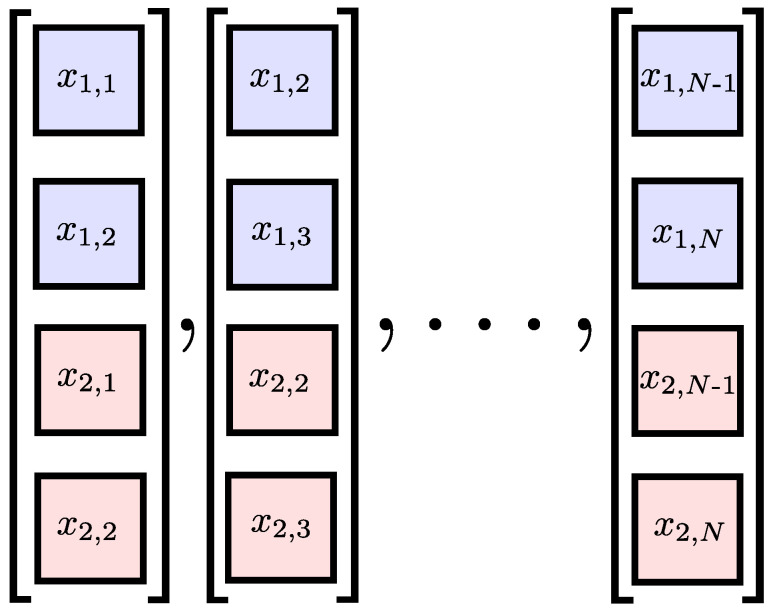
The *extended* multivariate delay vectors (DVs) of the multivariate DVs shown in Figure 2 using the proposed DV extension method (see Equation (9)).

At the core of the proposed algorithm, which addresses the above issues, is the manner in which multivariate DVs are extended to calculate the SE. To avoid the direct calculation of distances between the elements of different variates and DV misalignment, and to induce a desired mode-of-operation for correlated data, generating an extended set of multivariate DVs from those in Equation (4) is proposed in the following manner:

(9) $$\begin{array}{ccc} X_{M}^{e}\left(i\right) & = & [x_{1,i},\ldots ,x_{1,i+\left(m_{1}-1\right)\tau_{1}},x_{1,i+\left(m_{1}\right)\tau_{1}}\ldots ,\\ & & x_{k,i},\ldots ,x_{k,i+\left(m_{k}-1\right)\tau_{k}},x_{k,i+\left(m_{k}\right)\tau_{k}},\ldots ,\\ & & x_{P,i},\ldots ,x_{P,i+\left(m_{P}-1\right)\tau_{P}},x_{P,i+\left(m_{P}\right)\tau_{P}}] \end{array}$$

for i=1,…,N. For the bivariate case, the proposed DV extension is illustrated in Figure 7. We observe that distances are only calculated between elements of corresponding variates, making the approach perfectly suited for heterogeneous data.

**Figure 8:**
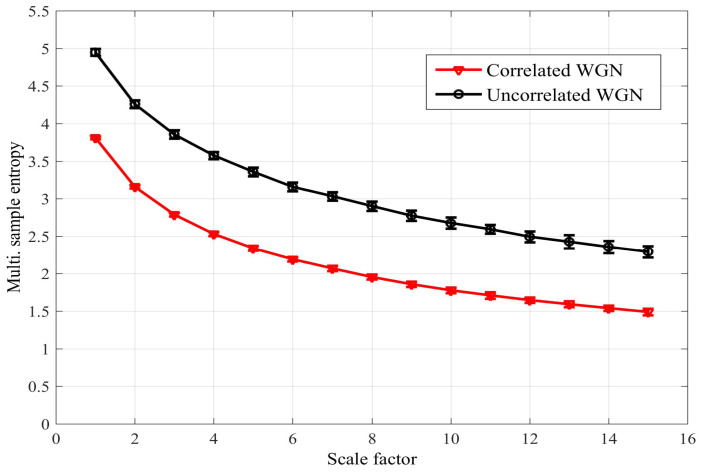
Multivariate sample entropy (mSE) estimate for bivariate white Gaussian noise (WGN), correlated and uncorrelated, using the proposed algorithm. The error bars denote ± standard deviation. We observe that increasing correlation reduces the SE (cf. Figure 4).

**Figure 9:**
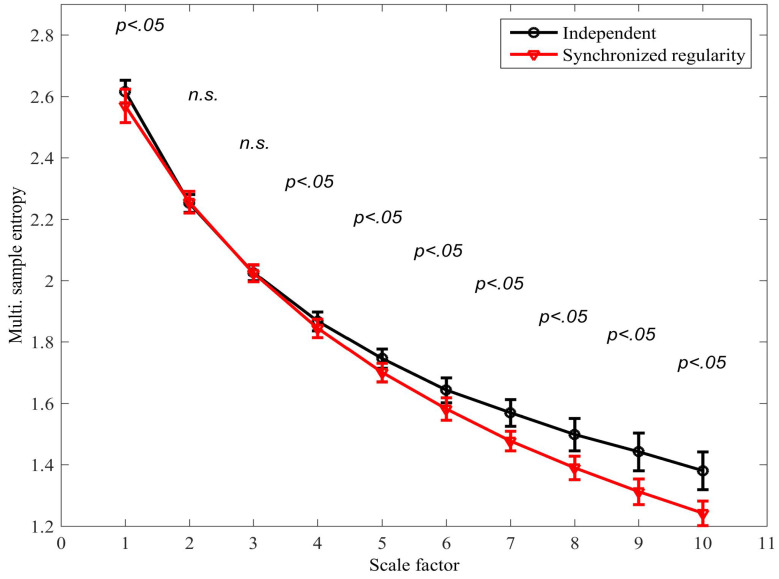
The multivariate multiscale sample entropy (mMSE) extension using the proposed algorithm for signals with (i) independent mixing parameters, and (ii) synchronized mixing parameters (see model described by Equations (6)–(8)). The error bars denote ± standard deviation. A two-tailed two-sample *t*-test was also applied to determine the degree of separation between the two scenarios at each scale factor; instances in which the distributions were found to overlap are denoted by “not-significant” (n.s.). Compare with the existing approaches in Figure 6.

Although the distances between DVs were not directly calculated between the elements of different variates, the estimation of multivariate SE on the basis of the proposed approach can still detect coupled regularity dynamics as well as existing methods, and in some instances, its performance enables better separation (see the following section). To support the claim of a desired mode-of-operation for correlated data, in the Appendix A, we present a model for the operation of the proposed multivariate algorithm in the presence of bivariate WGN; the model reveals how an increasing correlation between the variates reduces the multivariate SE. This is demonstrated in Figure 8 for the multiscale operation of the algorithm for bivariate WGN. The algorithm was also applied to time series with synchronized regularity between the variates generated using the same mixing model as before (see Equation (8) with β=−1.6); the results are shown in Figure 9. We note that, unlike the existing algorithm, a significant separation between the two time series also exists at the first scale factor (cf. Figure 4).

It is important to note an increase in the degree of coupling between the variates, either via correlation or synchronized regularity; in both cases this causes a decrease in the SE. It is natural to expect that real-world systems will exhibit different forms of coupling simultaneously and that an algorithm that behaves in a consistent way for each form of coupling is better equipped to model changes in multivariate regularity and complexity.

### 3.4. Multivariate Surrogates

For a given measure or index, it is common to employ signal surrogates to provide a baseline or reference value. Surrogates are a set of time series that share certain characteristics of the original signal but lack the property whose effect on the measure we wish to study [1]. For instance, in univariate sample analysis, it is common to generate surrogates that retain the spectrum shape of the original signal but that have randomized phase values, in this way creating similar signals with high irregularity and no structure (low complexity). In the same way, for greater insight, the SE of the original signal can be compared with the values obtained for the surrogates.

**Figure 10:**
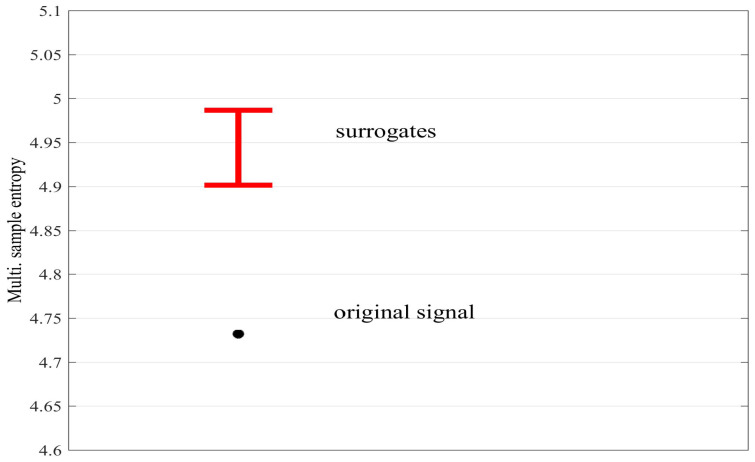
The multivariate sample entropy (mSE) for the original signal with correlated variates (black circle) and its multivariate surrogates (red error bars, which denote ± standard deviation). In the case of the surrogates, the interdependency (correlation) between the variates is removed and thus its average mSE value is higher, indicating the presence of coupled dynamics in the original signal.

For the multivariate case, it is desirable to remove any interdependencies between the variates in order to distinguish between within- and cross-channel regularity and complexity. We propose to utilize the multivariate surrogate approach used in previous synchronization measures, for example, in the study of asymmetry [21] and mutual information [22], whereby one of the variates is temporally shifted with respect to the other (random alignment). Figure 10 shows the mSE, obtained with parameters M=[1,1], τ=[1,1] and r=0.15, for correlated bivariate WGN (ρ=0.6; 5000 samples), as well as the average values obtained for 30 surrogates created by randomly shifting the second variate. We observe that the mSE of the original signal is significantly lower than that obtained for the surrogates, indicating the presence of coupled dynamics between the variates.

## 4. Simulations

The performance of the proposed algorithm was illustrated over the following case studies: (i) detecting joint regularity dynamics between recordings of cardiac function, and (ii) classifying multichannel recordings of brain function during wake and sleep states. In all cases, unless stated otherwise, the time series were scaled to zero mean and unit variance prior to mSE analysis.

### 4.1. Detecting Synchronized Cardiac Behaviour

We firstly considered a real-world synchronized regularity scenario. A healthy male subject (aged 32) performed a series of breathing exercises while his cardiac function was monitored via the electrocardiogram (ECG); electrodes were placed on the chest and ECG data were recorded using the gtec g.USBamp, a certified and U.S. Food and Drug Administration (FDA)-listed medical device, with a sampling frequency of 1200 Hz. The time difference between R peaks, sharp dominant peaks in the ECG waveform, was calculated and the R–R interval time series was generated using cubic spline interpolation at regular time intervals of 0.25 s.

**Figure 11:**
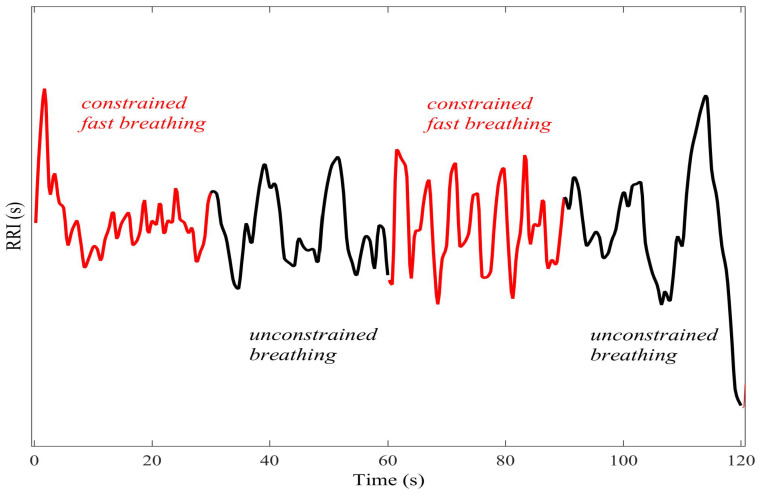
The R–R interval as the subject performed different breathing instructions (constrained/ unconstrained) every 30 s. As a result of the phenomenon of respiratory sinus arrhythmia, the breathing instructions induced periods of different regularity into the R–R interval.

For a given 30 s period, the instruction was either to (i) breathe normally (unconstrained), or (ii) breathe at a fixed rate of 15 breathing cycles (inhale/exhale) per minute aided by a metronome (constrained). The instruction was alternated from period to period. The periods of constrained breathing had the effect of inducing different regularity into the R–R interval through the phenomenon of respiratory sinus arrhythmia (RSA), the modulation of the cardiac output by the respiration effort [23]. Two 300 s trials were recorded; in the first, the subject started breathing in a constrained fashion, and in the second, the subject started breathing in an unconstrained fashion. Figure 11 shows a segment of the trial in which the subject started breathing in a constrained fashion. Prior to analysis, a high-pass filter with a cutoff frequency of 0.1 Hz was applied to the R–R interval.

**Figure 12:**
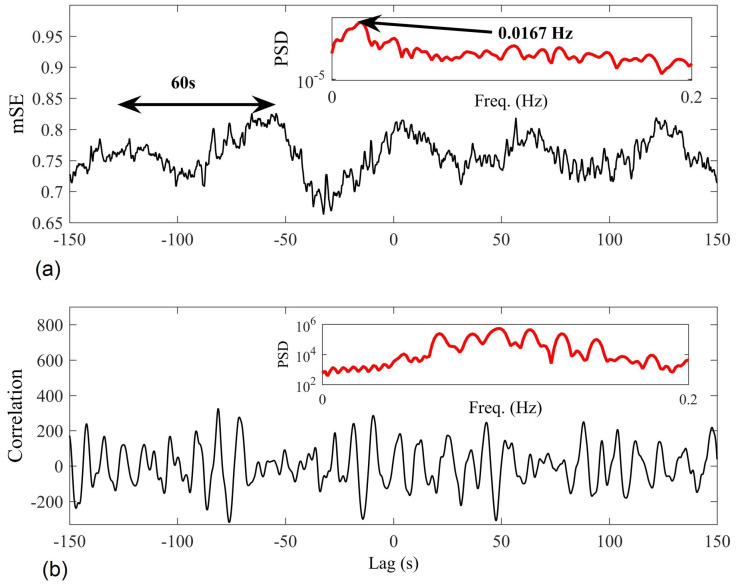
Multivariate sample entropy (mSE; (**a**)) and cross-correlation (**b**) results when applied to detect synchronized activity between two trials of R–R interval recordings. In the upper corner of each panel in red, the spectral analysis of each approach reveals the dominant synchronization period; only the mSE approach reveals synchronized regularity dynamics at 0.017 Hz (1/60).

Figure 12 compares the results of the proposed algorithm (M=[1,1], τ=[1,1], and r=0.4) with those obtained by cross-correlation, with the aim to determine the degree of synchronization between the two trials at different time-lags. Spectral analysis of the proposed mSE results revealed that synchronized regularity occurred at 60 s intervals (approximately 1/60 = 0.0167 Hz)—this clearly indicates joint regularity between the recording trials whenever the subject performed the same breathing instruction every 2 × 30 s. Furthermore, the approach revealed the correct time-lag between the trials, with the minimum mSE value occurring at lag −32 s. We observe that the cross-correlation approach was unable to reveal the synchronization in regularity; spectral analysis of the results showed no peak at 0.017 Hz.

### 4.2. Classifying Sleep States

The proposed algorithm was next applied to recordings of brain function during wake and sleep states. The data, electroencephalogram (EEG) recordings extracted from whole-night polysomnography recordings, were obtained from (http://www.tcts.fpms.ac.be/~devuyst/Databases/DatabaseSpindles/). Each 5 s segment of the EEG was independently annotated by an expert according to the Rechtschaffen and Kales criteria (wake; Rapid Eye Movement (REM); sleep stages S1, S2, S3 and S4). The task was to distinguish between the wake state and the state of slow-wave sleep (stages S3 and S4). Eight 30 min excerpts were available; however only four of these were considered. For two excerpts (2 and 7), there were insufficient wake periods (no sustained wake periods exceeding 1 min); for another excerpt (3), the sampling frequency was too low for meaningful analysis (50 Hz); and for another (8) there were no periods of slow-wave sleep. The excerpts analyzed were 1, 4, 5 and 6; excerpt 1 had a sampling frequency of 100 Hz, and the two channels of the EEG were obtained using the bipolar electrode configurations C3-A1 and FP1-A1; excerpts 4, 5 and 6 had a sampling frequency of 200 Hz, and the two channels of the EEG were obtained using the bipolar electrode configurations CZ-A1 and FP1-A1.

The multivariate SE was estimated (M=[1,1], τ=[1,1], and r=0.4) between corresponding EEG segments of length 2.5 s with a 50% overlap, with segments containing voltage values exceeding ±100 µV rejected to discard artefacts. The difference between the multivariate SE values for the states of wake and slow-wave sleep were determined using the Bhattacharyya distance, given by

(10) $$D=\frac{1}{4}\ln\left(\frac{1}{4}\left(\frac{\sigma_{w}^{2}}{\sigma_{s}^{2}}+\frac{\sigma_{s}^{2}}{\sigma_{w}^{2}}+2\right)\right)+\frac{1}{4}\left(\frac{{\left(\mu_{s}-\mu_{w}\right)}^{2}}{\sigma_{s}^{2}+\sigma_{w}^{2}}\right)$$

where $\mu_{w}$ and $\sigma_{w}$ denote the mean and standard deviation of the feature values for the wake state, and $\mu_{s}$ and $\sigma_{s}$ are the same values for the slow-wave sleep state. The degrees of separation using the existing and the proposed algorithm are shown in Table 1; we observe that for all excerpts, the degrees of separation between the states of wake and slow-wave sleep were greater using the proposed algorithm (the value of *D* is larger).

## 5. Conclusions

We have introduced an mSE algorithm that, unlike existing approaches, caters for heterogeneous data and yields improved insight into coupled regularity dynamics. This is exemplified, in part, by its consistent treatment of correlation and synchronized regularity, both of which exhibit low mSE values; this is in agreement with intuition concerning regularity and the complexity loss theory. Simulation results for the proposed algorithm reveal greater separation between neural data during different sleep stages. Unlike standard tools, which are invariably linear, it is also shown how the approach is sensitive to higher-order synchronization. Multivariate surrogate-generation techniques have been shown to enhance the significance of the results. The concept of synchronized regularity has been illuminated and a benchmark test proposed, which opens a new avenue of research in a number of applications.

## Figures and Tables

**Table 1:** The first two columns denote the degree of separation, calculated using the Bhattacharyya distance (*D*; see Equation (10)), in multivariate sample entropy (mSE) using the existing and proposed methods for bivariate electroencephalogram (EEG) recordings, between the states of wake (W) and slow-wave sleep (SWS), as annotated by an expert. The last columns denote the percentage of the entire recording annotated as wake (% W) and slow-wave sleep (% SWS).

	Existing	Proposed	% W	% SWS
Excerpt 1	D=1.28	D=1.66	14.5	19.9
Excerpt 4	D=1.99	D=3.54	27.1	6.5
Excerpt 5	D=1.10	D=2.06	10.8	28.6
Excerpt 6	D=1.48	D=1.61	3.3	32.3

**Figure A1:**
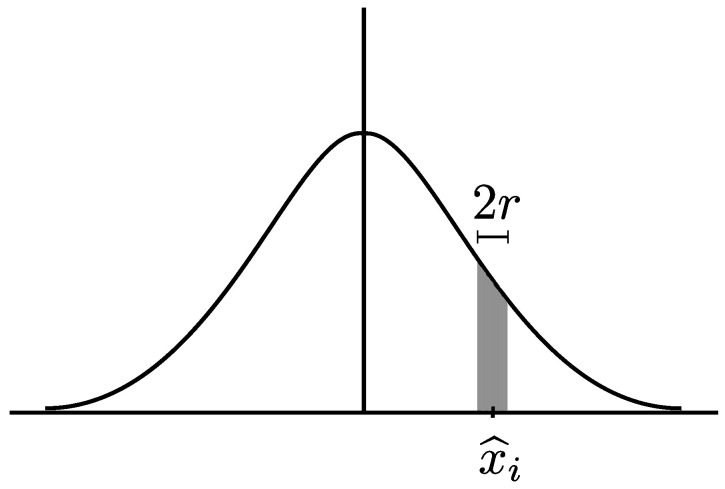
The area under the Gaussian curve, centred at ${\hat{x}}_{i}$, denotes the probability that the distance between this point and any other randomly selected point is less than or equal to *r*.

**Figure A2:**
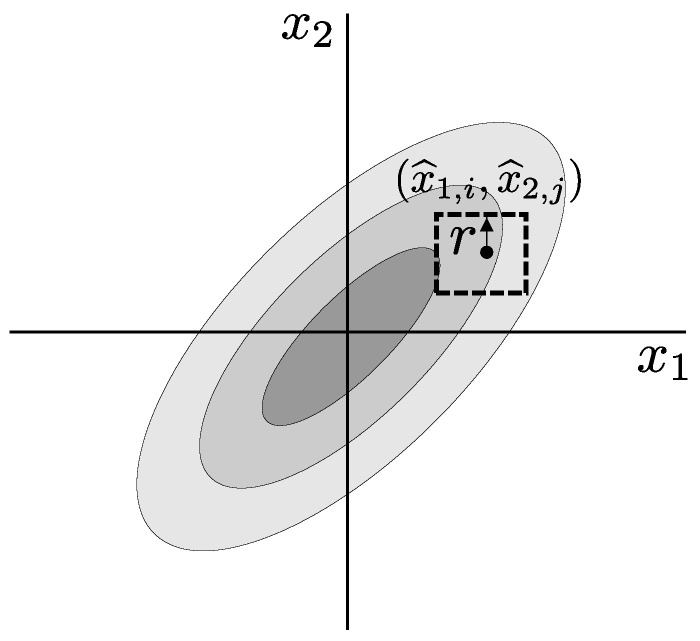
The volume under the surface of a normal bivariate distribution, centred at $({\hat{x}}_{1,i},{\hat{x}}_{2,j}$), denotes the probability that the distance between this point and any other randomly selected point is less than or equal to *r*.

**Figure A3:**
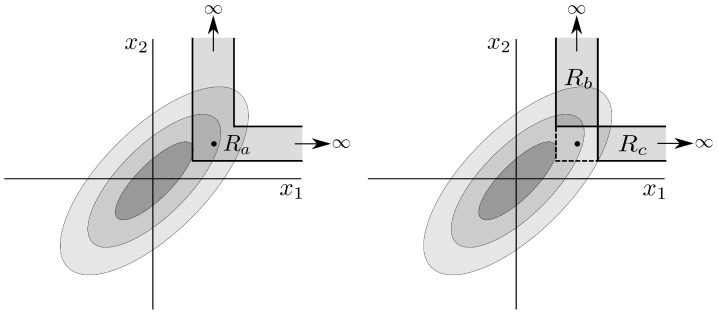
Approximating the bivariate cumulative distribution function at the locations shown can be used to approximate $P_{\left\{M=[1,1],r\right\}}$ (see Equation (A13)).

**Figure A4:**
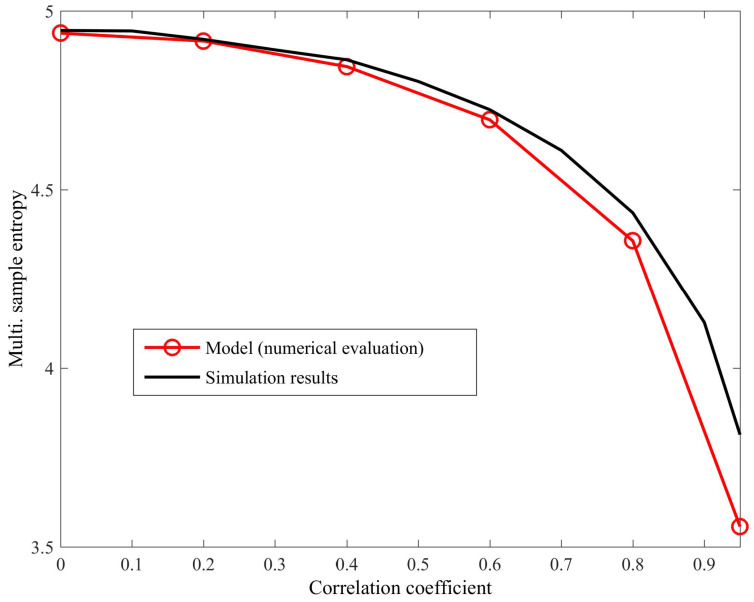
Numerical evaluation of the derived model (Equation (A13)) and simulation results for bivariate white Gaussian noise (WGN) with varying degrees of correlation (ρ). As expected, the accuracy of the model decreases for larger correlation values.

## Abbreviations

The following abbreviations are used in this manuscript:

AE	Approximate entropy
DV	Delay vector
ECG	Electrocardiogram
EEG	Electroencephalogram
mMSE	Multivariate multiscale sample entropy
MSE	Multiscale sample entropy
mSE	Multivariate sample entropy
RSA	Respiratory sinus arrhythmia
SE	Sample entropy
WGN	White Gaussian noise

## Appendix A. Multivariate SE for WGN

One of the key SE results is that for WGN. We here revisit the model for the operation of the univariate algorithm, presented in [10], and then derive a model for the case of bivariate WGN.

In the case of the univariate algorithm when m=1, the denominator in Equation (3) indicates the probability that the distance between two data points is less than or equal to *r*, that is, $|x_{i}-x_{j}|\leq r$, which we denote by

(A1) $$P_{\left\{m=1,r\right\}}=P(|x_{i}-x_{j}\left|\leq r\right)$$

The physical meaning of the numerator in Equation (3) is the probability that the pair-wise distance for DVs with embedding dimension m=2, the extended DVs, is less than or equal to *r*. As the elements in the DVs are independent random variables, the probability can be rewritten as follows (for simplicity, we here assume τ=1):

(A2) $$\begin{array}{cc} P_{\left\{m=2,r\right\}} & =P(|x_{i}-x_{j}\left|\leq r)\times P(\right|x_{i+1}-x_{j+1}\left|\leq r\right)\\ & =P_{\left\{m=1,r\right\}}\;P_{\left\{m=1,r\right\}} \end{array}$$

Generalizing this result for any embedding dimension, for univariate WGN, the SE is given by

(A3) $$\begin{array}{ccc} \mathrm{SE}(m,r) & = & \log_{e}\;\frac{P_{\left\{m,r\right\}}}{P_{\left\{m+1,r\right\}}}\\ & = & \log_{e}\;\frac{P_{\left\{m=1,r\right\}}\;P_{\left\{m=1,r\right\}}\cdots \;P_{\left\{m=1,r\right\}}}{P_{\left\{m=1,r\right\}}\;P_{\left\{m=1,r\right\}}\cdots \;P_{\left\{m=1,r\right\}}P_{\left\{m=1,r\right\}}}\\ & = & -\log_{e}P_{\left\{m=1,r\right\}} \end{array}$$

Thus, the SE for WGN is defined by $P_{\left\{m=1,r\right\}}$, which, for zero mean and unit variance, is given by

(A4) $$\begin{array}{c} P_{\left\{m=1,r\right\}}=P(|x_{i}-x_{j}\left|\leq r\right)=\\ \int_{-\infty }^{\infty }\left\{\int_{x_{i}-r}^{x_{i}+r}p\left(x_{j}\right)dx_{j}\right\}p\left(x_{i}\right)dx_{i}=\\ \frac{1}{2\pi }\int_{-\infty }^{\infty }\left\{\int_{x_{i}-r}^{x_{i}+r}e^{-x_{j}^{2}/2}dx_{j}\right\}e^{-x_{i}^{2}/2}dx_{i}=\\ \frac{1}{2\pi }\int_{-\infty }^{\infty }\left\{\mathrm{erf}\left(\frac{x_{i}+r}{\sqrt{2}}\right)-\mathrm{erf}\left(\frac{x_{i}-r}{\sqrt{2}}\right)\right\}e^{-x_{i}^{2}/2}dx_{i} \end{array}$$

The term within curly brackets {·} denotes the probability that a point at $x_{i}$ is within distance *r* of another randomly selected point. This is represented by the area under the normal curve in Figure A1. Numerically evaluating the model for r=0.15 gives 2.47, which matches the simulation result for the same parameters (see first scale factor in Figure 4b).

For the case of bivariate WGN, the proposed multivariate algorithm calculates the SE as

(A5) $$\mathrm{mSE}\left(M,r\right)=\log_{e}\;\frac{P_{\left\{M,r\right\}}}{P_{\left\{M^{e},r\right\}}}$$

where $P_{\left\{M,r\right\}}$ is the probability that the pair-wise distance for DVs with embedding dimension *M* is less than or equal to *r*, and $P_{\left\{M^{e},r\right\}}$ is the probability for the extended case as described by Equation (9). Similarly to the univariate case, independence between the multivariate DVs can be assumed, and the above can be rewritten as

(A6) $$\mathrm{mSE}\left(M,r\right)=-\log_{e}P_{\left\{M=[1,1],r\right\}}$$

where the probability is given by

(17) $$\begin{array}{c} P_{\left\{M=[1,1],r\right\}}=P(|x_{1,i}-x_{1,k}\left|\leq r,\right|x_{1,j}-x_{1,l}\left|\leq r\right)=\\ \int_{-\infty }^{\infty }\int_{-\infty }^{\infty }\left\{\int_{x_{1,k}=x_{1,i}-r}^{x_{1,i}+r}\int_{x_{1,l}=x_{1,j}-r}^{x_{1,j}+r}p\left(x_{1,k},x_{2,l}\right)\delta x_{1,k}\delta x_{2,l}\right\}\\ \;\;\;\;\;\;\;\;\;\;\;\;\;\;\;\;\;\;\;\;\;\;\;\;\;\;\;\;\times p\left(x_{1,i},x_{2,j}\right)\delta x_{1,i}\delta x_{2,j} \end{array}$$

for which, assuming variates with zero mean and unit variance, the joint probability is given by

(A8) $$\begin{array}{c} p(x_{1,i},x_{2,j})=\\ \frac{1}{2\pi \sqrt{1-\rho^{2}}}e^{\left(-\frac{1}{2(1-\rho^{2})}\left[x_{1,i}^{2}+x_{2,j}^{2}-2\rho x_{1,i}x_{2,j}\right]\right)} \end{array}$$

and ρ is the correlation coefficient. Similarly to the univariate case, the term within curly brackets {·} denotes the probability that a point at $x_{1,i},x_{2,j}$ is within distance *r* of another randomly selected bivariate point; this is represented by the volume under the bivariate surface in Figure A2.

Unlike the univariate case, however, a closed-form solution for the term enclosed in brackets does not exist for ρ≠0. However, it is possible to approximate the cumulative distribution function:

(A9) $$F\left({\hat{x}}_{1,i},{\hat{x}}_{2,j};\rho \right)=P\left(x_{1,k}>{\hat{x}}_{1,i},x_{2,l}>{\hat{x}}_{2,j}\right)$$

as follows [24]:

(A10) $$\begin{array}{c} F\left({\hat{x}}_{1,i},{\hat{x}}_{2,j}\right)\approx L\left({\hat{x}}_{1,i},{\hat{x}}_{2,j};\rho \right)=\\ F\left(-{\hat{x}}_{1,i}\right)[F\left\{\xi \left({\hat{x}}_{1,i},{\hat{x}}_{2,j};\rho \right)\right\}-\frac{1}{2}\rho^{2}{\left(1-\rho^{2}\right)}^{-1}\\ \xi \left({\hat{x}}_{1,i},{\hat{x}}_{2,j};\rho \right)p\left\{\xi \left({\hat{x}}_{1,i},{\hat{x}}_{2,j};\rho \right)\right\}\left[1+\mu \left({\hat{x}}_{1,i}\right)-\mu^{2}\left({\hat{x}}_{1,i}\right)\right]] \end{array}$$

where

(A11) $$\xi \left({\hat{x}}_{1,i},{\hat{x}}_{2,j};\rho \right)=\frac{\rho \mu \left({\hat{x}}_{1,i}\right)-{\hat{x}}_{2,j}}{\sqrt{\left(1-\rho^{2}\right)}}$$

and

(A12) $$\mu \left({\hat{x}}_{1,i}\right)=\frac{{\hat{x}}_{1,i}}{F(-{\hat{x}}_{1,i})}$$

Using this approximation, for which the accuracy decreases for increasing ρ, we can derive an approximation to the probability in Equation (A8) as

(A13) $$P_{\left\{M=[1,1],r\right\}}\approx R_{a}-\left(R_{b}+R_{c}\right)$$

where

(A14) $$\begin{array}{cc} R_{a}= & \\ & L\left({\hat{x}}_{1,i}-r,{\hat{x}}_{2,j}-r,\rho \right)-L\left({\hat{x}}_{1,i}+r,{\hat{x}}_{2,j}+r,\rho \right) \end{array}$$

(A15) $$\begin{array}{cc} R_{b}= & \\ & L\left({\hat{x}}_{1,i}-r,{\hat{x}}_{2,j}-r,\rho \right)-L\left({\hat{x}}_{1,i}+r,{\hat{x}}_{2,j}-r,\rho \right) \end{array}$$

(A16) $$\begin{array}{cc} R_{c}= & \\ & L\left({\hat{x}}_{1,i}-r,{\hat{x}}_{2,j}-r,\rho \right)-L\left({\hat{x}}_{1,i}-r,{\hat{x}}_{2,j}+r,\rho \right) \end{array}$$

The above expressions are illustrated in Figure A3. Combining the probability approximation in Equation (A13) with Equation (A6), we obtain a model that approximates the behaviour of the proposed algorithm for bivariate WGN for any correlation between the variates. A comparison of the numerical evaluation of the model and simulation results (averaged over 100 realizations of bivariate WGN; M=[1,1] and r=0.15; variate lengths of 3000 samples) for different correlation coefficient values (ρ) is shown in Figure A4. We observe that the model results match the simulation results, particularly for small values of ρ. However, the model is less accurate for larger values of ρ, as the accuracy of the approximation of the cumulative distribution function from [24], on which the model is based, decreases.
